# Plant health and its effects on food safety and security in a One Health framework: four case studies

**DOI:** 10.1186/s42522-021-00038-7

**Published:** 2021-03-31

**Authors:** David M. Rizzo, Maureen Lichtveld, Jonna A. K. Mazet, Eri Togami, Sally A. Miller

**Affiliations:** 1 Department of Plant Pathology, University of California-Davis, Davis, CA, USA; 2 Graduate School of Public Health, University of Pittsburgh, Pittsburgh, PA USA; 3 One Health Institute, School of Veterinary Medicine, University of California-Davis, Davis, CA, USA; 4 Department of Plant Pathology, The Ohio State University, 1680 Madison Ave, Wooster, OH 44691 USA

**Keywords:** Plant health, Aflatoxins, Pesticides, Food security, Food safety

## Abstract

Although healthy plants are vital to human and animal health, plant health is often overlooked in the One Health literature. Plants provide over 80% of the food consumed by humans and are the primary source of nutrition for livestock. However, plant diseases and pests often threaten the availability and safety of plants for human and animal consumption. Global yield losses of important staple crops can range up to 30% and hundreds of billions of dollars in lost food production. To demonstrate the complex interrelationships between plants and public health, we present four case studies on plant health issues directly tied to food safety and/or security, and how a One Health approach influences the perception and mitigation of these issues. Plant pathogens affect food availability and consequently food security through reductions in yield and plant mortality as shown through the first case study of banana Xanthomonas wilt in East and Central Africa. Case studies 2, 3 and 4 highlight ways in which the safety of plant-based foods can also be compromised. Case study 2 describes the role of mycotoxin-producing plant-colonizing fungi in human and animal disease and examines lessons learned from outbreaks of aflatoxicosis in Kenya. Plants may also serve as vectors of human pathogens as seen in case study 3, with an example of *Escherichia coli (E. coli)* contamination of lettuce in North America. Finally, case study 4 focuses on the use of pesticides in Suriname, a complex issue intimately tied to food security though protection of crops from diseases and pests, while also a food safety issue through misuse. These cases from around the world in low to high income countries point to the need for interdisciplinary teams to solve complex plant health problems. Through these case studies, we examine challenges and opportunities moving forward for mitigating negative public health consequences and ensuring health equity. Advances in surveillance technology and functional and streamlined workflow, from data collection, analyses, risk assessment, reporting, and information sharing are needed to improve the response to emergence and spread of plant-related pathogens and pests. Our case studies point to the importance of collaboration in responses to plant health issues that may become public health emergencies and the value of the One Health approach in ensuring food safety and food security for the global population.

## Background: plant health as part of one health

Although plant health is currently part of the definition of One Health [1], plants have typically not been well integrated into discussions of One Health approaches [2, 3]. However, plant health is vital to sustain human and animal health and a critical component of the complex interactions among the environment, humans, and animals. Recognizing the key role of plants in public health, the United Nations declared the year 2020 to be the International Year of Plant Health (IYPH) [4, 5]. The overarching purpose of the IYPH was to raise awareness of plant health and its effects on society [4]. Maintaining plant health has important consequences for human and animal health as an important driver of food security and safety, as a source of livelihoods in plant-based agriculture, as a source of pharmaceuticals, and as part of healthy environments [3, 6–9].

Plants provide over 80% of the food consumed by humans and are the primary source of nutrition for livestock [5]. Food security—the state of having reliable access to sufficient, safe, affordable, and nutritious food at all times—is necessary to have healthy and productive societies [7, 10]. Food security is also a crucial aspect of One Health and is a pillar of the United Nations Sustainable Development Goals (SDGs) [11, 12]. The UN definition of food security identified four key pillars: 1) availability, 2) access (both economic and socio-cultural), 3) utilization, including food preparation and safety, and 4) lastly the stability of these three pillars [13]. Food security thus reflects a complex value chain of production, food processing and distribution, and food access, beginning with plant health in the field. Employing a One Health approach to ensure the safety and continuity of this value chain will result in the protection and advancement of public health.

Plant diseases and pests influence the availability and safety of plants for human and animal consumption, reduce crop yield and detrimentally affect quality [9, 14]. Measures to prevent or treat diseases, including application of pesticides, may adversely impact the health of agricultural workers and consumers, as well as drive the development of antimicrobial and antifungal resistance in pathogens [15, 16]. In addition, food plants may serve as carriers of human pathogens and harmful microbial-based toxins. For example, foodborne illnesses pose a serious global burden on human health, reportedly affecting 600 million people or 33 million Disability Adjusted Life Years (DALYs) in a single year [17]. Although international food standards, such as the Codex Alimentarius, are implemented to protect consumers’ health and fair trade, foodborne illnesses continue to affect high-, middle-, and low-income countries around the world [17, 18]. Plants are important origins of foodborne outbreaks, including fresh vegetables and fruits irrigated with, washed with, or exposed to water and soil contaminated with pathogens of animal or human origin. More than half (51%) of outbreak-associated illnesses in the US were traced to plant-foods over a 10-year period, higher than any other food commodity, such as meat, fish, and dairy products [19]. Additionally, antibiotic resistant bacteria and resistance genes originating from animal feces can also contaminate fresh produce and pose health risks for humans [20]. Therefore, a key aspect of food security is timely and effective management of plant pathogens and pests and other microbes associated with plants that can cause foodborne illnesses, often disproportionately impacting the most vulnerable and health disparate populations locally and globally.

The emergence of new variants of pathogens and pests, as well as the expansion of the geographic range of known ones, can cause significant disruption in food production and pose a burden on the global economy. Global yield losses of important staple crops to pathogens and pests can range up to 30% with estimated costs to the global economy due to lost food production in the hundreds of billions of dollars [7]. Effective pest and disease management approaches, including pesticide management strategies, are required to successfully prevent and mitigate these issues. Recognition of and action to address the need for quantification of crop losses and their impact on humans, plants, animals, and land use are critically important [2, 10, 21]. Traditional surveillance strategies are often expensive and associated with a delay in problem recognition and access to actionable data. The lack of time-sensitive responses to foodborne outbreaks negatively impacts public health and the food service industry.

To show the complex interrelationships between plants and public health, and to demonstrate the value of the One Health approach, we review four cases studies. One study shows the relationship between plant health and food security. Two case studies involving a naturally-occurring pathogen (one plant-based and one animal-based) show the relationship between plant food safety and human health. The final case study involving a man-made toxin represents a study of both food security and food safety. Through these case studies, we examine challenges and opportunities moving forward for mitigating negative public health consequences and ensuring health equity.

## Case studies

### Case study 1: plant pathogens and food availability: banana Xanthomonas wilt in east and Central Africa, 2001-present (Fig. 1; Fig. 2-1)

Based on currently available data, up to 30% of global staple food crops are lost annually due to plant pests, including diseases, insects, and weeds, but excluding abiotic factors such as drought, excessive water, or poor soils [7]. When diseases severely affect staple crops in low income or under-resourced regions of the world, food availability is threatened, potentially resulting in malnutrition and population-based famine in severe cases. In addition, loss of income from cash crops sold by small commercial farms can have a cascading effect, exacerbating poverty among populations who depend upon farmers to purchase goods and services from the rural non-farm sector.

**Fig. 1:**
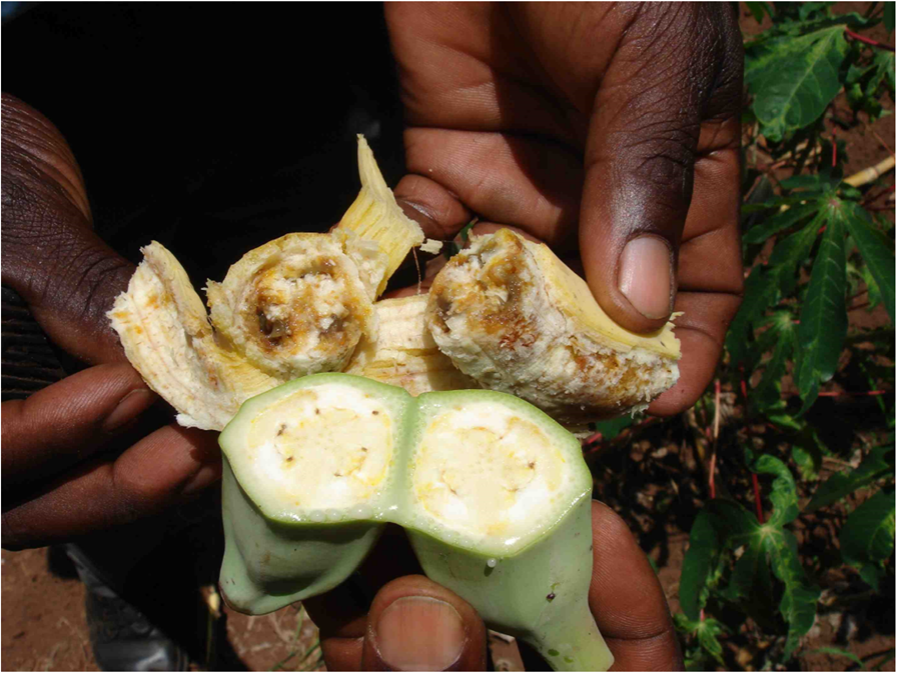
Rotting banana fruit caused by the bacterial phytopathogen *Xanthomonas campestris* pathovar *musacearum* in Uganda. The disease (banana Xanthomonas wilt) also causes wilting and death of banana plants and significant reductions in availability of this staple food in East and Central Africa (source S. Miller)

**Fig. 2:**
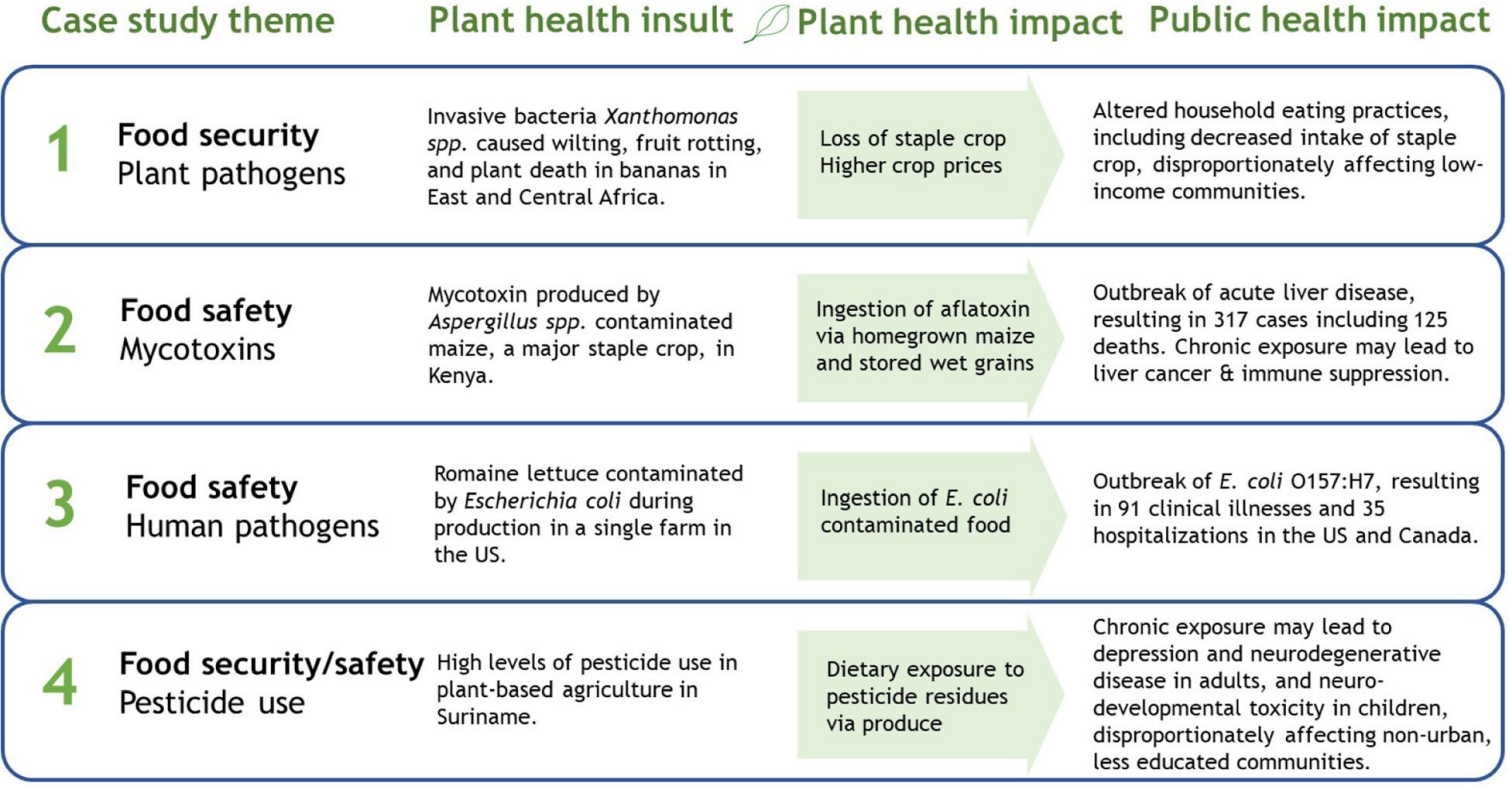
Case studies and linkages to One Health as discussed in the text. The studies illustrate different examples of the interconnectedness of plant, animal and human health, and the negative consequences of plant health problems to public health

Bananas (*Musa* spp.), including dessert banana, plantain, and cooking banana, comprise the eighth most important food crop in the world and the fourth most important in low- and middle-income countries in terms of gross production value [22]. Bananas are a staple crop and important source of protein, starch, vitamins, and minerals in East and Central Africa, ranging from 20% of household food consumption each day in Uganda to 80% in Rwanda [23]. Bananas historically had been among the least expensive of the staple crops to produce [24]. They have multiple uses in crop production systems, such as cycling carbon and soil nutrients, preventing soil erosion, and providing shade for understory crops [23]. Additionally, bananas are a source of food for livestock and used for the production of goods such as baskets, carpets, and shoes [23].

Banana Xanthomonas wilt (BXW) is an invasive bacterial disease first observed in Uganda in 2001, where it developed into a severe epidemic within 4 years of emergence and spread to the Democratic Republic of the Congo, Rwanda, Kenya, Tanzania, and later to Burundi [22]. BXW is caused by *Xanthomonas campestris* pathovar *musacearum*, a gram-negative rod-shaped bacterium that enters the plant through wounds or natural openings, colonizes the plant’s vascular system, and causes wilting, fruit rotting, and plant death [22]. The disease is spread by planting infected banana pseudostems, by various insects that visit the male flowers, by wind-driven rain, and by using contaminated cutting tools [22].

During the peak of the 2000–2010 epidemic in heavily affected areas of Tanzania, Burundi, and Rwanda, there were significant harmful effects on food production, availability, and subsequently, household consumption practices [25]. For example, the production of banana beer and juice declined approximately 60%, and the number of banana bunches sold and consumed declined 35 and 25% respectively, compared to pre-BXW levels. Importantly, the BXW epidemic greatly affected the availability of bananas, because the price of banana bunches increased by 46% compared to pre-BXW levels. As a result of reduced banana availability and higher prices, households coped by eating fewer meals than usual, eating smaller meals than needed, or substituting bananas with other available food items, such as maize, cassava, or sweet potatoes. Increased food prices have a disproportionate influence on low income households, often already struggling to meet basic needs. Loss of bananas from cropping systems may also result in damage to the environment (soil erosion) and reduced crop productivity (loss of shade) [26]. Long-term impacts of BXW epidemics on human nutrition and health, poverty, the environment and cultural practices in East and Central Africa have not yet been determined.

There are no effective antibiotics or other pharmaceutical treatments available for BXW. However, the disease can be managed by adoption of specific farming practices, including removing the male flower buds to reduce insect transmission of the pathogen, removing or burning affected plant pseudostems, decontaminating farm tools after each use, and using disease-free planting materials [26]. The cost and complexity of these disease management practices pose barriers to adoption by farmers [27]. Deployment of BXW-resistant varieties would be a significant step forward in managing this disease locally and reducing its spread. However, banana breeding is extremely slow and difficult, and sources of resistance to BXW have not been found in cultivated banana. *Musa balbisiana* is the only known source of resistance but is not preferred for breeding due to genome configuration differences [28]. Genetically engineered (GE) bananas resistant to BXW have been developed and tested extensively in field trials [22, 29]. In Uganda, which has been particularly hard-hit by BXW, it has been estimated that adoption of GE bananas could benefit farmers by $15 million and consumers by $10 million annually and could result in 55,000 people escaping poverty each year. BXW-resistant GE banana varieties may be available for distribution by 2023, if an appropriate biosafety regulatory system is in place. However, for Uganda and other countries affected by BXW, political considerations based on public perceptions of GMO foods may delay or even stop the implementation of the necessary regulatory systems [30]. In the meantime, increased adoption of BXW cultural management practices can be better facilitated by effective training programs such as farmer field schools and refining disease management strategies into more feasible and easy-to-implement recommendations. These include limiting removal of BXW-affected plants to dry periods when the disease is least likely to spread, covering cut plant stems with soil and sterilizing tools in fire pits within banana fields [26]. Furthermore, leveraging advances in technology to track transmission patterns by using innovative citizen science strategies as well as collaboration between scientists and farmers to improve training, can accelerate progress toward prevention of BXW. Specifically, citizen science and information communication technologies can accelerate the identification of new outbreaks, information sharing can enable rapid decision making among farmers, and enhanced connectivity among stakeholders can create networks for collective action [31].

### Case study 2: food safety and mycotoxins: aflatoxicosis outbreak in Kenya, 2004–2005 (Fig. 2-2)

Aflatoxin B_1_ is a type of mycotoxin produced by *Aspergillus flavus* and *A. parasiticus* [32]. Mycotoxins, which are small molecular-weight fungal metabolites, are produced on a wide array of food plants and are toxic to animals and humans [33]. Aflatoxins can contaminate human foods such as cereals, roots, nuts, and pulses under favorable conditions such as high temperatures, high humidity, and drought stress, which lead to plant colonization by the *A. flavus* and *A. parasiticus* molds [32]. In 2004–2005, aflatoxin contamination of maize—a major staple food in Kenya—was found to be the cause of a severe outbreak of acute liver disease in eastern Kenya, resulting in 317 cases, including 125 deaths [33]. The primary risk factor for aflatoxicosis in this event was the consumption of homegrown maize, followed by storage of wet grain in the home [33]. Aflatoxin B_1_ concentrations in stored maize in affected households were up to 50 times the limit prescribed for food in Kenya [33]. Chronic aflatoxin B_1_ contamination is a risk factor for acute liver damage, which may lead to chronic illnesses including liver cancer and immune system suppression [32].

The toxicity, morbidity, and potentially lethal effects of aflatoxin, as observed in the Kenya outbreak, highlight the significance of aflatoxin as an important public health challenge. Humans are exposed to aflatoxins through contaminated food crops or by consuming products from animals that have been exposed to contaminated feed. Aflatoxin contamination threatens the health and wellbeing of already vulnerable populations, such as children and individuals with hepatitis B virus (HBV) and hepatitis C virus (HCV) infections [32]. Children are especially susceptible to aflatoxins and can suffer short- and long-term effects such as malnutrition and stunting [32]. Additionally, chronic exposure to aflatoxins disproportionately affects low-resourced populations, with an estimated 5 billion people in low- and middle-income countries (LMICs) at risk of chronic exposure to aflatoxins [34].

Chronic exposure to aflatoxins has detrimental effects on animal health and can cause growth inhibition and immune suppression [32]. Consequently, health risks to animals and humans alike and compounding impacts on livelihoods result when aflatoxicosis is not prevented. Limited availability of food, lack of regulatory systems for monitoring and controlling aflatoxin, and environmental conditions that favor fungal development in crops are some of the common factors that increase the likelihood of aflatoxin poisoning [32]. Therefore, preventing and mitigating aflatoxin poisoning requires employing a One Health approach to protect human, plant, and animal health.

Ultimately, prevention and mitigation of aflatoxin contamination of food and feed, particularly in LMICs that often lack the expertise and infrastructure to effectively prevent and interdict aflatoxin contamination, require multi-pronged, economically-feasible, integrated approaches supported by private and public sector entities. For example, the appropriate and recommended use of irrigation and insecticides during the pre-harvest period and hand sorting of grains and effective rodent control during the post-harvest period could mitigate risks of aflatoxin contamination. Increasing awareness of the burden of aflatoxin exposure on public health is critical to encourage implementation of these strategies. In response to the deadly 2004–2005 outbreak in Kenya, Kenyan government agencies investigated the cause and established the National Food Safety Coordinating Committee in 2006, active at the policy level and coordinating mycotoxin testing in food and feed, inspection, enforcement, education, and program monitoring and evaluation [35]. A holistic, coordinated approach including plant, animal, and public health research and practice is necessary to address the gaps in knowledge, technology and education to prevent aflatoxicosis. These include insufficient documentation of human exposure, lack of measurements of economic impacts of aflatoxin contamination throughout various value chains and analyses of long-term impacts of aflatoxin mitigation approaches, inadequate sampling of grains on smallholder farms and storage facilities, lack of consumer awareness of the attributes associated with aflatoxin contamination and absence of economic incentives for the production and/or marketing of low-aflatoxin grain [35, 36].

### Case study 3: human pathogens associated with plants and food safety: *E. coli* O157:H7 outbreak caused by romaine lettuce in the United States and Canada, 2018–2019 (Fig. 2-3)

Between October 2018 and January 2019, a foodborne outbreak of Shiga-toxin producing *E.coli* O157:H7 (STEC) resulted in 91 illnesses and 35 hospitalizations, including four cases of hemolytic uremic syndrome (HUS) but no deaths, in multiple areas of the United States (US) and Canada [37, 38]. Fortunately, the outbreak was detected in its early stages by US and Canadian surveillance systems, including FoodNet and PulseNet, and on November 1, 2018 the US Food and Drug Administration (FDA), CDC, Canadian Food Inspection Agency (CFIA), and Public Health Agency of Canada initiated a multi-agency outbreak investigation. On November 20, FDA issued a public health advisory warning to consumers not to eat romaine lettuce until further notice, a bold and atypical advisory against a type of produce without identifying its farm of origin. In Canada, CFIA advised industry not to import, distribute, or sell romaine lettuce during the investigation. Ultimately, trace back of suspected food ingredients, field visits, and laboratory testing, including whole-genome sequencing, determined that the cause of the outbreak was in fact romaine lettuce produced on a farm in Santa Barbara, California, whose irrigation system was implicated as the contamination source for *E. coli* O157:H7. Genetic characterization of the pathogens revealed that the DNA footprints of the *E. coli* strains in this outbreak were genetically closely linked among cases, as well as related to a previous *E. coli* outbreak that affected the US and Canada in December 2017 [37–39]. By early January 2019, despite its extensive geographical spread, the outbreak was contained and declared over within 11 to 14 weeks since the recognition of illness in the initial cases in both the US and Canada.

By employing One Health approaches to food surveillance, public health, and animal health, and taking rapid actions as demonstrated during this outbreak, public health officials will be better able to understand the source of foodborne illnesses, rapidly enabling and informing prevention and mitigation measures for future outbreaks. It is important to consider that the intestinal tracts of healthy ruminant animals are reservoirs of *E. coli* O157:H7, and cattle feces are believed to be a major source for human illness [40]. Shedding of *E. coli* O157:H7 by cattle is influenced by seasonality, food production strategies, and life stage of cattle. In addition, the pathogen can persist in the environment, such as in water troughs, in animal feces that are not removed expeditiously, and on feedlots [41]. In this outbreak, there was no conclusive evidence that the water was contaminated from domestic ruminant feces. However, the final investigation highlighted that ruminant intestinal tracts are well-established reservoirs for *E. coli* O157:H7. Wildlife and humans can also be sources of bacterial contamination of the food supply, and investigators noted “evidence of extensive wild animal activity, including waterfowl, rodents, coyotes, etc., and animal burrows near the contaminated reservoir sediment,” likely warranting intensive exploration in future outbreaks, including water supplies. In California, this and other foodborne outbreaks have highlighted the importance of complying with, and accelerated the implementation of, local and national produce safety practices, such as the Produce Safety Rule under the Food Safety Modernization Act of the FDA which came into effect in 2016 [42, 43]. Farmers are required to reduce the likelihood of direct or indirect contamination of produce with wildlife fecal material through soil, water, vehicles, and other means of transmission and have taken an active role in preventing foodborne illnesses as integral stakeholders for protecting public health.

The successful rapid containment of this large-scale STEC outbreak can be attributed to early detection of the event through robust surveillance systems, swift multi-agency coordination with an employment of the One Health approach, use of whole genome sequencing for *E. coli* characterization, and timely and appropriate issuance of a broad public health advisory. Plant, environmental, animal, and human health experts will likely continue to be challenged by the burden of foodborne illnesses, which should be addressed by continuing to coordinate multi-agency prevention, detection, response, and containment strategies; incorporating state-of-the art technologies to identify pathogens; and balancing the benefits of protecting the health of populations with the economic cost of issuing prompt public safety advisories.

### Case study 4: pesticide use in plant-based agriculture and food security and safety in Suriname, 2010–2015 (Fig. 2–4; Fig. 3)

Suriname, a middle-income country located on the northeastern coast of South America, has one of the highest pesticide use rates per area crop land in the Caribbean (8.8 kg/ha). Pesticides are intended for preventing, destroying, repelling, or mitigating any pest [44]. They can be classified according to the target organism (most commonly: insecticides, herbicides, and fungicides), or molecular structure (such as insecticides categorized as organophosphates, carbamates, organochlorines, pyrethroids, and neonicotinoids). The term pesticide in this case study will refer to chemical pesticides that are used for agricultural purposes.

**Fig. 3:**
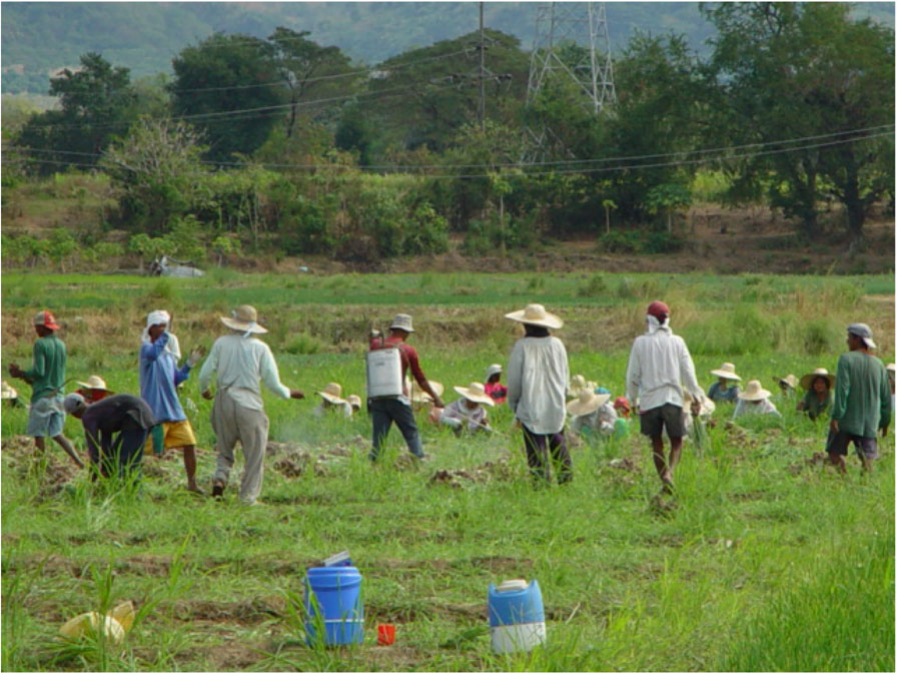
Manual application of pesticides (center) with potential exposure of workers during manual crop maintenance operations in Southeast Asia (Source: S. Miller)

Agriculture is a developing sector in Suriname, which contributes approximately 9% to the Gross Domestic Product (GDP) and employs 17% of the population [45, 46]. Screening data from the Dutch Food and Consumer Product Safety Authority (NVWA) from 2010 to 2015 consistently showed pesticide residues in crops imported from Suriname [47]. The Caribbean Consortium for Research in Environmental and Occupational Health (CCREOH) is examining the association of pesticide exposure to birth outcomes in 1000 mother/child dyads [48]. CCREOH’s preliminary environmental assessment showed pesticide residues in Surinamese produce, including the insecticides endosulfan and lindane in the leafy vegetable *Xanthosoma brasiliense* (tannia). According to an interviewer-assisted dietary survey, which was administered to assess dietary exposure to pesticides in Surinamese women (including 696 pregnant women), women living in non-urban districts and less educated women were more likely to have a higher tannia intake rate compared to those living in urban districts and women who received higher levels of education [47]. This disparity in exposure to tannia illustrates how pesticide use can have inequitable consequences for food safety based on region and education level.

Pesticides play an important role in food security by protecting crops from pests and diseases, leading to improved productivity. However, the misuse of pesticides may lead to residues in produce, potentially compromising food safety [49]. In addition, the use of banned pesticides continues to be a problem in low-income countries. Due to financial constraints and lack of policy and enforcement, less favorable (older, more toxic and environmentally persistent) pesticides are being used in these countries [50].

Chronic low-level pesticide exposures, such as through diet, are harmful to human health and have been associated with depression and neurodegenerative disease in adults [51, 52]. Furthermore, exposure during gestation and the early postnatal period has been associated with a lower birth weight, decreased gestational age, and neuro-developmental toxicity that can lead to motor- and neurocognitive developmental delays in children [53, 54]. In addition, the presence of pesticide residues in plants and the environment has been linked to the emergence of antimicrobial resistant organisms [15]. Recently, the use of triazole fungicides in certain horticultural systems in Europe has been linked to the emergence of azole-resistant environmental isolates of *Aspergillus fumigatus* and subsequent fatal human aspergillosis cases [55]. Especially in the case of Suriname, the greenest country in the world with biodiverse and unique flora and fauna, it is important to consider the possible negative consequences of pesticides on wildlife, such as loss of species and declines in diversity [56–58].

Appropriate use of pesticides is important for safeguarding food security, food safety, and health equity. While pesticide use is highly regulated in high income countries, there is an urgent need in LMICs to formulate policies on pesticide residues in plants and the environment, such as monitoring and reporting levels of pesticide residues. In Suriname, the development and implementation of national pesticide policies are limited, and the country does not monitor pesticide residues in crops. Although there is currently no policy on the emergence and spread of resistance in plant pathogens associated with pesticides, guidance exists for developing comprehensive action plans to address these potential threats.

Training farmers to use pesticides correctly and screening pesticide residues in crops are pivotal to reducing human risk of pesticide exposure. In addition, approaches to reduce the use of pesticides should be implemented. A well-known strategy is Integrated Pest Management (IPM), which prioritizes the use and integration of multiple cultural, biological, and host resistance strategies, while reducing pesticide use, to manage pests and diseases of plants and animals [59]. IPM is geared toward improving economic benefits of production systems and reducing human health risks and adverse environmental effects of pesticide use. Ultimately, implementation of IPM, development and enforcement of international recommendations and national policies, and equipping of farmers with the knowledge and means to use pesticides appropriately will minimize pesticide residues in food and the environment and enable economically sustainable food production while reducing adverse health effects in people.

## Conclusions

Threats to plant health pose challenges to population health, productivity, and prosperity across the globe. Efforts to protect plants from emerging and endemic pathogens and pests help to not only increase food security and safety to ensure healthy lives, but also to alleviate poverty, promote equity, confront the impact of climate change, protect the environment, boost economic development, and strengthen global partnerships. Establishing a much closer partnership among advocates for One Health, including experts in sustainable agriculture, and public health practitioners will lead to promoting a safe, sustainable, and nutritious diet for families worldwide.

The case studies presented above demonstrate how management practices aimed at reducing crop losses and ensuring food safety would benefit from employment of a One Health approach. Outbreaks of emerging pathogens can be mitigated by mobilizing experts and resources from all arms of One Health to elevate integrated research and development in human, animal, and plant health. For example, protecting bananas from the harmful effects of banana Xanthomonas wilt and alleviating the shortage of food caused by the disease involves a framework that highlights the interaction and interdependence of physical and socio-cultural factors across all levels of a health problem [60]. This involves effective integrated training on individual and organizational levels; collaboration with plant, environmental, and animal health specialists on the interpersonal level; and implementation of feasible policies on the community and society levels. Similar approaches can be utilized in ensuring food safety, as evidenced in the cases of aflatoxicosis, *E. coli*, and pesticide use. The 2004 case of aflatoxicosis in Kenya resulted in an intervention on the societal and policy levels with positive effects observed by individuals, communities and organizations. Suriname’s case of pesticide use and safety concerns exhibits a need for societal policy interventions that lead to positive cascading effects on the economy and other parts of society.

The relationship between plant health and human health is especially important in public health and illustrates a need for research specifically focused on the direct and indirect effects of compromised plant health to human populations. Research and development that allows for inclusion of multiple potential causes for public health concern, including plant diseases and pests that endanger human and animal health and wellbeing, is vital for holistically preventing and mitigating the effects of public health threats. To successfully and effectively protect plant health and address food security, there will need to be a stronger regulatory framework, effective surveillance and monitoring systems, feasible disease management practices, and effective training of food production professionals in protecting plant, animal, environmental, and human health. Our case studies also point to the importance of interagency coordination in facilitating rapid responses to public health emergencies, benefits of technological advances that facilitate data sharing, and the value of the One Health approach in ensuring food safety and food security for the global population.

## Data Availability

Not applicable.

## Abbreviations

BXW: Banana Xanthomonas wilt
CCREOH: Caribbean consortium for research in environmental and occupational health
CFIA: Canadian Food Inspection Agency
GDP: Gross domestic product
HCV: Hepatitis C virus
HUS: Hemolytic uremic syndrome
IPM: Integrated pest management
IYPH: International year of plant health
LMIC: Low- and middle-income countries
SDGs: Sustainable development goals
STEC: Shiga-toxin producing *E. coli*
UN: United Nations

## References

[1] Centers for Disease Control and Prevention (CDC). One Health Basics. https://www.cdc.gov/onehealth/basics/. Accessed 5 Feb 2021.

[2] Destoumieux-Garzon D. et al. The One Health concept: 10 years old and a long road ahead. Frontiers in Veterinary Science. 2018; 5: article 14. 10.3389/fvets.2018.00014 PMC5816263 29484301

[3] Fletcher J, Franz D, LeClerc JE (2009). Healthy plants: necessary for a balanced “one health” concept. Vet Ital.

[4] United Nations General Assembly. Resolution adopted by the general assembly on 20 December 2018*,* 2019.

[5] Food and Agriculture Organization of the United Nations (FAO). International year of plant health – protecting plants, Protecting life. 2020. http://www.fao.org/plant-health-2020.

[6] Boa E, Danielsen S, Haesen S. Better together: identifying the benefits of a closer integration between plant health, agriculture and one health. In: Zinsstag J, Schelling E, Waltner-Toews D, Whittaker M, Tanner M, editors. One Health: The Theory and Practice of Integrated Health Approaches. Wallingford: CABI; 2015. p. 258–72.

[7] Savary S, Willocquet L, Pethybridge SJ, Esker P, McRoberts N, Nelson A (2019). The global burden of pathogens and pests on major food crops. Nat Ecol Evol.

[8] Scholthof KG (2003). One foot in the furrow: linkages between agriculture, plant pathology, and public health. Ann Rev Public Health.

[9] Strange RN, Scott PR (2005). Plant disease: a threat to global food security. Ann Rev Phytopathol.

[10] International Food Policy Research Institute. Food Security. https://www.ifpri.org/topic/food-security. Accessed 5 Feb 2021.

[11] Choffnes ER, Relman DA, Olsen LA, Hutton R, Mack A (2012). Improving food safety through a one health approach. Forum on microbial threats, Institute of Medicine. The.

[12] United Nations. Sustainable Development Goals. https://www.un.org/sustainabledevelopment/sustainable-development-goals/. Accessed 5 Feb 2021.

[13] Food and Agriculture Organization of the United Nations (FAO). World summit on Food security, Rome, 2009; www.fao.org/wsfs/world-summit/en/.

[14] Savary S, Bregaglio S, Willocquet L, Gustafson D (2017). Crop health and its global impacts on the components of food security. Food Secur.

[15] Ramakrishnan B, Venkateswarlu K, Sethunathan N, Megharaj M (2019). Local applications but global implications: can pesticides drive microorganisms to develop antimicrobial resistance?. Sci Total Environ.

[16] Fisher MC, Hawkins NJ, Sanglard D, Gurr SJ (2018). Worldwide emergence of resistance to antifungal drugs challenges human health and food security. Science.

[17] Foodborne Disease Burden Epidemiology Reference Group 2007-2015. WHO Estimates of the Global Burden of Foodborne Diseases. Geneva: World Health Organization; 2015. pp. 265.

[18] Food and Agriculture Organization of the United Nations (FAO)/World Health Organization (WHO). About Codex Alimentarius; http://www.fao.org/fao-who-codexalimentarius/about-codex/en/. Accessed 5 Feb 2021.

[19] Painter JA, Hoekstra RM, Ayers T (2013). Attribution of foodborne illnesses, hospitalizations, and deaths to food commodities by using outbreak data, United States, 1998-2008. Emerg Infect Dis.

[20] US Food and Drug Administration. Investigation Summary: Factors Potentially Contributing to the Contamination of Romaine Lettuce Implicated in the Fall 2018 Multi-State Outbreak of E coli O157:H7. 2019; https://www.fda.gov/food/outbreaks-foodborne-illness/investigation-summary-factors-potentially-contributing-contamination-romaine-lettuce-implicated-fall.

[21] Wilkinson K, Grant WP, Green LE (2011). Infectious diseases of animals and plants: an interdisciplinary approach. Phil Trans R Soc B.

[22] Tripathi L, Mwangi M, Abele S, Aritua V, Tushemereirwe WK, Bandyopadhyay R (2009). Xanthomonas wilt: a threat to banana production in east and Central Africa. Plant Dis.

[23] Geberewold AZ (2019). Review on impact of banana bacterial wilt (Xhantomonas campestris pv. Musacerum) in East and Central Africa. Cogent Food Agric.

[24] Chandler S, Gowen S (1995). The nutritional value of bananas. Bananas and Plantains.

[25] Nkuba J, Tinzaara W, Night G (2015). Adverse impact of Banana Xanthomonas wilt on farmers livelihoods in eastern and Central Africa. African J Plant Sci.

[26] Shimwela MM, Ploetz RC, Beed FD (2016). Banana xanthomonas wilt continues to spread in Tanzania despite an intensive symptomatic plant removal campaign: an impending socio-economic and ecological disaster. Food Secur.

[27] Di Cori V, Kikulwe E, Kozicka M, Gotor E (2018). Understanding the economic impact of BXW and its management practices in east and Central Africa.

[28] Nakato GV, Christelova P, Were E, Nyine M, Coutinho T, Dolezel J, Uwimana B, Swennen R, Mahuku G (2019). Sources of resistance in *Musa* to *Xanthomonas campestris* pv*. musacearum*, the causal agent of banana xanthomonas wilt. Plant Pathol.

[29] Tripathi L, Atkinson H, Roderick H, Kubiriba J, Tripathi JN (2017). Genetically engineered bananas resistant to Xanthomonas wilt disease and nematodes. Food Energy Secur.

[30] Kikulwe EM, Falck-Zepeda JB, Oloka HK, Chambers JA, Komen J, Zambrano P, Wood-Sichra U, Hanson H. Benefits from the adoption of genetically engineered innovations in the Ugandan banana and cassava sectors: an *ex ante* analysis. IFPRI discussion paper 1927. Washington, DC: International Food Policy Research Institute (IFPRI). 2020. https://doi.org/10.2499/p15738coll2.133716.

[31] McCampbell M, Schut M, Van den Bergh I (2018). Xanthomonas wilt of Banana (BXW) in Central Africa: opportunities, challenges, and pathways for citizen science and ICT-based control and prevention strategies. NJAS Wageningen J Life Sci.

[32] Ogodo AC, Ugbogu OC (2016). Public health significance of aflatoxin in food industry – a review. European journal of clinical and biomedical. Sciences.

[33] Lewis L, Onsongo M, Njapau H (2005). Aflatoxin contamination of commercial maize products during an outbreak of acute aflatoxicosis in eastern and Central Kenya. Environ Health Perspect.

[34] Williams JH, Phillips TD, Jolly PE, Stiles JK, Jolly CM, Aggarwal D. Human aflatoxicosis in developing countries: a review of toxicology, exposure, potential health consequences, and interventions. Am J Clin Nutr. 2004;80:1106–22. 10.1093/ajcn/80.5.1106. 10.1093/ajcn/80.5.1106 15531656

[35] Mutegi CK, Cotty PJ, Bandyopadhyay R (2018). Prevalence and mitigation of aflatoxins in Kenya (1960-to date). World Mycotoxin J.

[36] Hoffman V, Mutiga SK, Harvey JW, Nelson RJ, Milgroom MG. Observability of food safety losses in maize: evidence from Kenya. Food Policy 2020; https://doi.org/10.1016/j.foodpol.2020.101895

[37] Government of Canada. Public health notice - outbreak of E. coli infections linked to romaine lettuce. 2019; https://www.canada.ca/en/public-health/services/public-health-notices/2018/outbreak-ecoli-infections-linked-romaine-lettuce.html.

[38] Centers for Disease Control and Prevention (CDC). Outbreak of E. coli infections linked to romaine lettuce. 2019; https://www.cdc.gov/ecoli/2018/o157h7-11-18/index.html.

[39] Centers for Disease Control and Prevention (CDC). Whole genome sequencing. https://www.cdc.gov/ncezid/dfwed/keyprograms/tracking-foodborne-illness-wgs.html. Accessed 5 Feb 2021.

[40] Mead PS, Griffin PM (1998). Escherichia coli O157:H7. Lancet.

[41] Hancock DD, Besser TE, Rice DH, Ebel ED, Herriott DE, Carpenter LV (1998). Multiple sources of Escherichia coli O157 in feedlots and dairy farms in the northwestern USA. Prev Vet Med.

[42] California Department of Food and Agriculture Inspection Services Division, CDFA Produce Safety Program. https://www.cdfa.ca.gov/producesafety/. Accessed 16 January 2021.

[43] Food and Drug Administration, Food Safety Modernization Act (FSMA), FSMA Final Rule on Produce Safety. Updated 3 September 2020. https://www.fda.gov/food/food-safety-modernization-act-fsma/fsma-final-rule-produce-safety. Accessed 16 January 2021.

[44] US EPA. Basic information about pesticide ingredients. https://www.epa.gov/ingredients-used-pesticide-products/basic-information-about-pesticide-ingredients. Updated 2019.

[45] Ministry of Agriculture, Animal husbandry and fisheries. The National Agricultural Innovation Strategy of the Republic of Suriname. 2013; http://extwprlegs1.fao.org/docs/pdf/sur171413.pdf.

[46] Derlagen C, Barreiro-Hurlé J, Shik O (2013). Agricultural Sector Support in Suriname.

[47] Abdoel Wahid F, Wickliffe J, Wilson M (2017). Presence of pesticide residues on produce cultivated in Suriname. Environ Monit Assess.

[48] Lichtveld MY, Hawkins WB, Ouboter PE (2016). A one health approach to interdict environmental health threats in Suriname. Ann Glob Health.

[49] Carvalho FP (2006). Agriculture, pesticides, food security and food safety. Environ Sci Policy.

[50] Ecobichon DJ. Pesticide use in developing countries. Toxicology 2001;160:27–33. doi: https://doi.org/10.1016/S0300-483X(00)00452-2. 10.1016/s0300-483x(00)00452-2 11246121

[51] London L, Beseler C, Bouchard MF (2012). Neurobehavioral and neurodevelopmental effects of pesticide exposures. Neurotoxicology.

[52] Beard JD, Umbach DM, Hoppin JA (2014). Pesticide exposure and depression among male private pesticide applicators in the agricultural health study. Environ Health Perspect.

[53] Burns CJ, McIntosh LJ, Mink PJ, Jurek AM, Li AA (2013). Pesticide exposure and neurodevelopmental outcomes: review of the epidemiologic and animal studies. J Toxicol Environ Health B Crit Rev.

[54] Rauch SA, Braun JM, Barr DB (2012). Associations of prenatal exposure to organophosphate pesticide metabolites with gestational age and birth weight. Environ Health Perspect.

[55] European Centre for Disease Prevention and Control (2013). Risk assessment on the impact of environmental usage of triazoles on the development and spread of resistance to medical triazoles in Aspergillus species.

[56] REDD+ Suriname. https://surinameredd.org/en/reddplus-suriname/. Updated 2020.

[57] Relyea RA (2005). The impact of insecticides and herbicides on the biodiversity and productivity of aquatic communities. Ecol Appl.

[58] Henriques W, Jeffers R, Lacher T, Kendall R (1997). Agrochemical use on banana plantations in Latin America: perspectives on ecological risk. Environ Toxicol Chem.

[59] US Environmental Protection Agency USEP. Integrated Pest Management (IPM) Principles. https://www.epa.gov/safepestcontrol/integrated-pest-management-ipm-principles. Accessed 5 Feb 2021.

[60] US Dept Health and Human Services, National Institutes of Health. Theory at a glance. A guide for health promotion practice. 2005. http://www.sbccimplementationkits.org/demandrmnch/wp-content/uploads/2014/02/Theory-at-a-Glance-A-Guide-For-Health-Promotion-Practice.pdf

